# Therapeutic pCRISPRi Delivery to Lung Squamous Cell Carcinoma by Combining Nanobubbles and Ultrasound

**DOI:** 10.3390/pharmaceutics17081053

**Published:** 2025-08-13

**Authors:** Taiki Yamaguchi, Yoko Endo-Takahashi, Takumi Amano, Arina Ihara, Tetsushi Sakuma, Takashi Yamamoto, Takuya Fukazawa, Yoichi Negishi

**Affiliations:** 1 Department of Drug Delivery and Molecular Biopharmaceutics, School of Pharmacy, Tokyo University of Pharmacy and Life Sciences, 1432-1 Horinouchi, Hachioji 192-0392, Tokyo, Japan; y171184@toyaku.ac.jp (T.Y.);; 2 Laboratory of Genome Editing Breeding, Graduate School of Agriculture, Kyoto University, 448-5 Kajiicho, Kamigyo-ku, Kyoto-shi 602-0841, Kyoto, Japan; sakuma.tetsushi.5k@kyoto-u.ac.jp; 3 Genome Editing Innovation Center, Hiroshima University, 3-10-23 Kagamiyama, Higashi-Hiroshima 739-0046, Hiroshima, Japan; tybig@hiroshima-u.ac.jp; 4 Department of General Surgery, Kawasaki Medical School, 2-6-1 Nalasange, Kita-ku, Okayama 700-8505, Okayama, Japan; fukazawat@med.kawasaki-m.ac.jp

**Keywords:** nanobubbles, ultrasound, gene delivery, CRISPR interference, squamous cell carcinoma, cancer gene therapy, ΔNp63, SOX2

## Abstract

**Background/Objectives**: Lung squamous cell carcinoma (SCC), a major subtype of non-small cell lung cancer, remains a significant clinical challenge due to a scarcity of actionable molecular targets and the limited effectiveness of current targeted therapies. Emerging treatment strategies inhibit the gene expression of lineage survival oncogenes such as ΔNp63 and SOX2. CRISPR interference (CRISPRi) is a promising method to downregulate these genes; however, the efficacy depends on effective delivery. Here, we focused on the delivery system using nanobubbles (NBs) and ultrasound (US) for site-specific CRISPRi delivery to SCC. We evaluated the therapeutic efficacy of plasmid-based CRISPRi (pCRISPRi) targeting SOX2 or ΔNp63 using intratumoral pCRISPRi/NBs injections followed by US. **Methods**: A mixture of NBs and pCRISPRi was injected directly into the tumors and exposed to US-induced cavitation to facilitate pCRISPRi uptake. Tumor volume was measured every other day, and apoptosis was assessed by TUNEL assay. **Results**: In a lung SCC xenograft model, NBs/US-mediated pCRISPRi delivery induced apoptosis and significantly suppressed tumor growth. **Conclusions**: These findings suggest that US-guided, NB-facilitated delivery of pCRISPRi can locally suppress lineage survival oncogenes and trigger tumor cell death, representing a promising targeted therapy for lung SCC. Additionally, this platform could be adapted to other cancers by targeting alternative factors.

## 1. Introduction

Lung cancer remains the leading cause of cancer-related mortality worldwide and is classified into small-cell and non-small-cell lung cancer (NSCLC), with NSCLC accounting for 85–90% of cases [1,2]. Among NSCLC subtypes, squamous cell carcinoma (SCC) comprises approximately 20%, second only to adenocarcinoma. Unlike adenocarcinoma, SCC often lacks actionable driver mutations (e.g., EGFR, ALK), limiting the success of targeted therapies [3,4]. While immune checkpoint inhibitors (e.g., pembrolizumab) have improved outcomes in PD-L1-high patients and are now standard first-line treatments alongside platinum-based chemotherapy [5], many patients experience limited responses and serious immune-related adverse effects.

Gene therapy provides an alternative strategy. Chimeric antigen receptor (CAR)-T-cell therapy has shown success in hematologic malignancies and is being explored for solid tumors, including lung cancer. However, its use in SCC is limited by the lack of tumor-specific surface antigens required for safe and effective CAR design [6]. This highlights the need for flexible gene regulation systems.

The CRISPR-Cas9 system enables precise genome editing [7]. Catalytically deactivated Cas9 (dCas9) fused with transcriptional regulators and guided by gRNAs enables transcriptional repression (CRISPR interference, CRISPRi) or activation (CRISPR activation, CRISPRa) of target genes [8]. Compared with knockout approaches or RNAi, CRISPRi offers safer and more precise gene regulation with lower off-target effects [9].

Previously, we developed a vector system that co-expresses Cas9 and multiple gRNAs for efficient genome editing [10]. We also showed that ΔNp63, a lineage survival oncogene in SCC, is effectively repressed via CRISPRi, inhibiting SCC cell growth in vitro and in vivo [11].

Despite the promise of CRISPRi, effective localized delivery in vivo remains a challenge. Viral vectors offer high transduction but carry risks, such as immunogenicity and insertional mutagenesis, and limited packaging capacity. In contrast, non-viral vectors such as lipid nanoparticles are safer and have greater design flexibility but are less efficient in vivo [12,13].

To improve specificity and efficacy, drug delivery systems increasingly use external stimuli to control release. Ultrasound (US), light, and magnetic fields enhance payload delivery through increased membrane permeability or triggered cargo release [14,15,16]. US, in particular, is noninvasive, deeply penetrative, and clinically translatable [17]. When paired with US contrast agents (e.g., microbubbles, MBs), US enhances membrane permeability in the limited area of US irradiation and selective intracellular uptake of therapeutics, including drugs, genes, and nucleic acids [18,19,20,21]. In fact, it has been applied to the delivery strategy of CRISPR systems [22,23,24].

Nanobubbles (NBs) have also attracted increasing attention for use as both US contrast agents and US-mediated drug delivery. The mechanistic distinction between MBs and NBs remains an active area of investigation. Stride et al. have highlighted potential limitations of NBs, suggesting that observed bioeffects attributed to NBs may result from contaminating MBs present in the solution [25]. However, the actual outcomes likely depend on various factors, including the target tissue, US conditions, the material and concentration of the bubbles, and the US device used. Several studies have supported the unique potential of NBs for tumor-targeted delivery. For instance, Goertz et al. reported that intravenously administered MBs can be fragmented into smaller NBs upon US irradiation, which subsequently extravasate from the vasculature and accumulate in the tumor interstitium [26]. Similarly, submicron phase-change droplets possess a higher potential to reach perivascular and extravascular tumor regions via enhanced permeability and retention (EPR) effects [27]. Both reports suggest that NBs may reach deeper tumors and exert their therapeutic effects in close proximity to tumor cells. In the context of our current study, vascular extravasation is not a contributing factor, as an intratumoral injection route was employed. However, the favorable dispersion of NBs within tumor tissue may also be useful for local administration. Furthermore, their high structural stability may reduce vulnerability to tumor-associated interstitial pressure, thereby supporting consistent delivery performance.

In our previous work, we developed NBs—based on PEG liposomes, ultrafine bubbles meeting ISO definitions—for biomedical applications [28,29]. A combination of NBs and US has been shown to work as a gene delivery tool via local administration [30,31,32]. Accentually, these NBs could reach into the tumor interstitium by local administration and cavitate under US, temporarily permeabilizing membranes to allow for the uptake of co-administered plasmid DNA (pDNA) [33]. As NBs are capable of widely dispersing within the tumor parenchyma upon local injection, they offer a strategy for enhancing gene delivery efficiency throughout tumor tissue.

In this study, we employed NBs/US delivery of dCas9–KRAB with multiplex gRNAs targeting lineage survival oncogenes in SCC xenografts. In addition to ΔNp63, we also targeted SOX2—another lineage survival oncogene in SCC [34,35,36].

## 2. Materials and Methods

### 2.1. Materials

The lipids 1,2-dipalmitoyl-sn-glycero-3-phosphocholine (DPPC) and N-(carbonyl-methoxy polyethylene glycol 2000)-1,2-distearoyl-sn-glycero-3-phosphoethanolamine (DSPE-PEG2000) were purchased from NOF Corporation (Tokyo, Japan). The anionic lipid, 1,2-dipalmitoyl-sn-glycero-3-phospho-(1′-rac-glycerol) (DPPG) was obtained from Avanti Polar Lipids (Alabaster, AL, USA). Perfluoropropane gas was supplied by Takachiho Chemical Industrial Co., Ltd. (Tokyo, Japan).

### 2.2. Preparation of NBs

Anionic liposomes were prepared using the reverse-phase evaporation method, as previously described [37]. DPPC and DSPE-PEG2000 were dissolved in chloroform. DPPG was dissolved in a mixture of chloroform, methanol, and HEPES-buffered saline (HBS; 150 mM NaCl, 10 mM HEPES, pH 7.0) in a 60:30:5 *v*/*v*/*v* ratio. Lipids (DPPG:DPPC:DSPE-PEG2000 = 50:44:6) were mixed with chloroform, diisopropyl ether, and HBS (1:1:1, *v*/*v*/*v*), sonicated, and evaporated. The organic solvent was completely removed, and the liposomes were resized to adjusted to <200 nm using extrusion equipment and a sizing filter (Whatman Plc., Kent, UK). The liposomes were then sterilized by filtration using a 0.45 μm syringe filter (Asahi Techno Glass Co., Chiba, Japan). Lipid concentration was determined using a phosphorus assay based on the Fiske method [37,38]. A calibration curve was generated using KH_2_PO_4_ aqueous solutions (0–4 mM). For the assay, the liposome suspension was treated with perchloric acid and nitric acid, incubated at 200 °C for 1 h, cooled, and mixed with ammonium molybdate tetrahydrate, hydrochloric acid, and freshly prepared ascorbic acid. The samples were incubated at 60 °C for 2.5 min, and absorbance was measured at 820 nm. The assay was based on the reduction of phosphate–molybdate complexes by ascorbic acid to form a colored compound.

To generate NBs, 0.8 mL of liposome suspension (1 mg/mL total lipid) was placed in 2 mL sterilized vials, filled with perfluoropropane gas, sealed, and pressurized with an additional 3 mL of perfluoropropane. The vials were sonicated in a bath sonicator (40 kHz, Bransonic M2800; Branson Ultrasonics Co., Danbury, CT, USA) for 5 min. Zeta potential was measured using a Zetasizer Nano ZSP (Malvern Instruments, Ltd., Malvern, UK). NB number concentration and mean size were determined via nanoparticle tracking analysis (Viewsizer 3000; HORIBA, Ltd., Kyoto, Japan).

### 2.3. Cell Culture

EBC2 lung SCC cells were generously provided by Dr. Katsuyuki Kiura (Department of Respiratory Medicine, Okayama University Graduate School of Medicine and Dentistry, Okayama, Japan). Cells were cultured in RPMI 1640 medium (FUJIFILM Wako Pure Chemical Corporation, Osaka, Japan) supplemented with 10% heat-inactivated fetal bovine serum (Nichirei Biosciences Inc., Tokyo, Japan), 100 U/mL penicillin, and 100 μg/mL streptomycin at 37 °C in a humidified atmosphere.

### 2.4. Preparation of Tumor-Bearing Mice

BALB/c nu/nu mice were subcutaneously injected in the left flank, with 2 × 10^6^ EBC2 cells suspended in 100 μL of cold Hank’s Balanced Salt Solution, as described previously [11,35]. Once tumors reached 90–110 mm^3^, the mice were randomized to each group; this was designated as day 1 for subsequent experiments. Mice were assigned to the treatment groups based on tumor volume. Tumor volume was calculated using the following formula:

$$\mathrm{Tumor}\;\mathrm{volume}=0.5\times \mathrm{length}\times {\mathrm{width}}^{2}$$

### 2.5. US Imaging

Mice bearing EBC2 tumors were anesthetized and intratumorally injected with NBs (20 μg, total lipid in 60 μL HBS). Tumors were imaged using an Aplio80 US diagnostic system (Toshiba Medical Systems, Tokyo, Japan) with a 12 MHz wideband transducer. Contrast harmonic imaging was performed using a mechanical index of 0.27.

### 2.6. pDNA

The plasmid pcDNA3-Luc (Promega, Madison, WI, USA) expresses firefly luciferase under the CMV promoter.

Plasmid-based CRISPRi (pCRISPRi)ΔNp63A/B and pCRISPRiSOX2A/B were constructed using a Multiplex CRISPR/Cas9 Assembly Kit (#1000000055) and dCas9 Accessory Pack (#1000000062) from Addgene (Cambridge, MA, USA), respectively, as previously described [10,11,39]. The gRNA template sequences were as follows: ΔNp63A_s: CACCGATTCATATTGTAAGGGTCT; ΔNp63A_as: AAACAGACCCTTACAATATGAATC; ΔNp63B_s: CACCGAAATCCTGGAGCCAGAAGAA; ΔNp63B_as: AAACTTCTTCTGGCTCCAGGATTTC; SOX2A_s: CACCGAAGAGAGTGTTTGCAAAAG; SOX2A_as: AAACCTTTTGCAAACACTCTCTTC; SOX2B_s: CACCGTTTGCTGCCTCTTTAAGACT; and SOX2B_as: AAACAGTCTTAAAGAGGCAGCAAAC. Each pDNA was dissolved in TE buffer (Nacalai Tesque Inc., Kyoto, Japan) and stored as a stock solution at a concentration of 2 μg/μL.

### 2.7. Luciferase Assay

NBs (20 μg, total lipid in 60 μL HBS) and pDNA (10 μg in 5 μL TE buffer) were intratumorally injected into the EBC2 tumor, followed by US irradiation (frequency: 1 MHz, duty cycle: 50%; intensity: 2.0 W/cm^2^, duration: 2 min) using the Sonitron 2000 (NEPA GENE, Co., Chiba, Japan).

Luciferase expression was evaluated 1 day post-injection transfection using an in vivo imaging system. Tumors were collected and homogenized in lysis buffer (0.1 M Tris–HCl, pH 7.8; 0.1% Triton X-100; 2 mM EDTA), and luciferase activity was measured with a luciferase assay system (Promega) and Synergy HTX plate reader (BioTek Japan, Tokyo, Japan). Results are reported as relative light units per milligram of protein.

### 2.8. Real-Time PCR

Tumors were treated with pCRISPRi (10 μg in 5 μL TE buffer) and NBs (20 μg, lipid in 60 μL HBS), followed by US (frequency: 1 MHz, duty cycle: 50%; intensity: 2.0 W/cm^2^, duration: 2 min) on days 1, 3, and 5. Total RNA from the tumors harvested on day 6 was obtained by using RNAiso Plus (Takara Bio, Otsu, Japan). A 1 μg quantity of total RNA was used for reverse transcription. Reverse transcription was performed using ReverTra Ace qPCR RT Master Mix (Toyobo, Osaka, Japan) according to the manufacturer’s instructions. The specific probes for *CDKN1A* (Hs00355782_m1), *ΔNp63* (Hs00978339_m1), *SOX2* (Hs01053049_s1), and *Glyceraldehyde-3-phosphate dehydrogenase* (*GAPDH*) (Hs03929097_g1) were derived from the commercially available TaqMan Gene Expression Assays (Thermo Fisher Scientific Inc., Waltham, MA, USA). The real-time PCR reactions were performed using TaqMan Fast Advanced Master Mix for qPCR (Thermo Fisher Scientific Inc.) and a real-time RT-PCR system (CFX96; Bio-Rad Laboratories, Inc., Hercules, CA, USA). The number of cycles for the amplification plot to reach the threshold limit (Ct value) was used for quantification. GAPDH was used as endogenous control.

### 2.9. Terminal Deoxynucleotidyl Transferase-Mediated dUTP–Biotin Nick End Labeling (TUNEL) Staining

Tumors were treated with pCRISPRi (10 μg in 5 μL TE buffer) and NBs (20 μg, lipid in 60 μL HBS), followed by US (frequency: 1 MHz, duty cycle: 50%; intensity: 2.0 W/cm^2^, duration: 2 min) on days 1, 3, and 5. On day 6, tumors were harvested, fixed in 4% formaldehyde overnight, incubated in 20% sucrose overnight, embedded in OCT, and frozen at −80 °C. Five-micrometer sections were prepared with a cryostat. Apoptosis was detected via a DeadEnd Colorimetric TUNEL System (Promega), following the manufacturer’s instructions. Sections were examined under a fluorescence microscope (BZ8100; KEYENCE, Osaka, Japan), and apoptotic areas were quantified using ImageJ (version 1.54g).

### 2.10. Animals

Six-week-old BALB/c nu/nu mice were obtained from Japan SLC Inc. (Shizuoka, Japan). Anesthesia was administered using a combination of 0.75 mg/kg medetomidine, 4.0 mg/kg midazolam, and 5.0 mg/kg butorphanol. All animal procedures were approved by the Tokyo University of Pharmacy and Life Sciences Animal Care Committee and complied with ARRIVE guidelines.

### 2.11. Statistical Analysis

Data are presented as mean ± standard deviation (*n* = 3–5). Differences between two groups were analyzed using Student’s *t*-test. In case of multiple group comparisons, statistical significance was determined using one-way analysis of variance (ANOVA) and followed by Tukey’s post hoc test. Data were considered statistically significant at *p* < 0.05.

## 3. Results

### 3.1. NBs Preparation and Characterization

**Figure 1:**
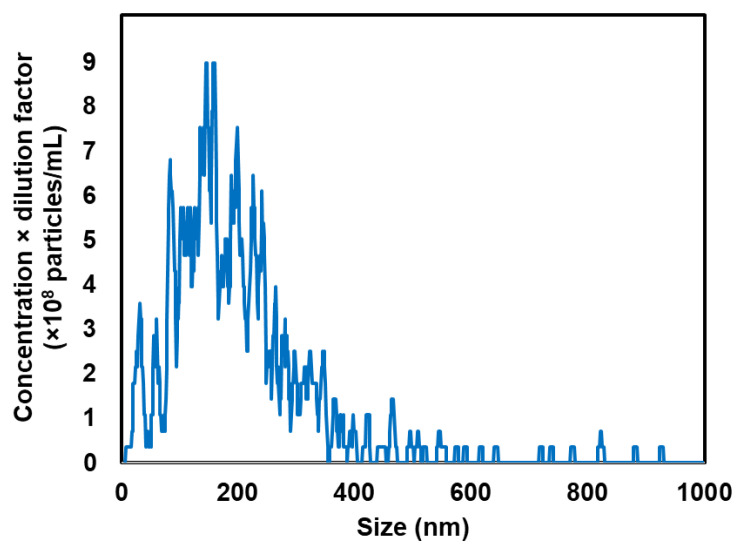
Particle size distribution measured via nanoparticle tracking analysis (Viewsizer 3000).

We previously reported that NBs containing anionic lipids are stable [37]. Therefore, anionic lipid-containing NBs were utilized in this study. NBs stability, which drives gene transfer, to resist high internal tumor pressure and enhanced the gene transfer efficacy. NBs were analyzed using nanoparticle tracking analysis and dynamic light scattering (Figure 1, Table 1, Video S1).

### 3.2. Intratumoral Distribution of NBs via US Imaging

**Figure 2:**
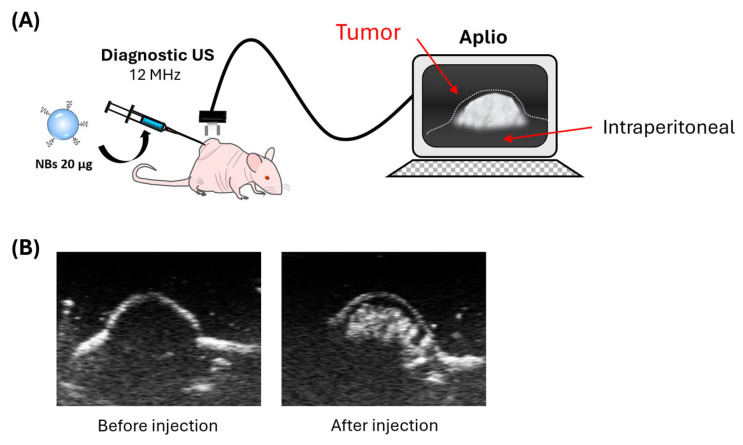
Contrast harmonic imaging following intratumoral NB administration was performed using an Aplio80 US diagnostic system (frequency: 12 MHz; depth: 0.75 cm; mechanical index: 0.27). Samples containing 20 μg NBs were administered intratumorally. (**A**) Schematic of US imaging using diagnostic US. (**B**) Contrast images of the tumor.

NBs serve as US contrast agents. Exploiting this property, we evaluated NB distribution within the tumors following intratumoral administration (Figure 2A). As shown in Figure 2B, NBs were broadly distributed throughout the tumor.

### 3.3. pDNA Delivery into Tumors via NBs and US Irradiation

**Figure 3:**
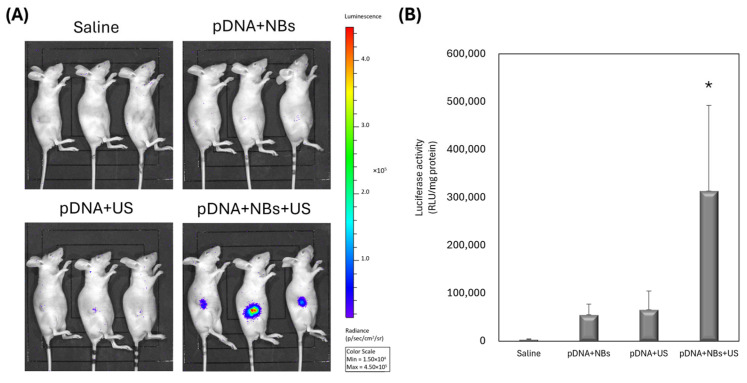
Intratumoral gene delivery using NBs and US irradiation (frequency: 1 MHz; duty cycle: 50%; intensity: 2.0 W/cm^2^; duration: 2 min). Samples containing 20 μg NBs and 10 μg pDNA were administered intratumorally, followed by US. (**A**) Luciferase imaging 24 h post-injection. (**B**) Luciferase activity in tumor homogenates. Data represent mean ± SD (*n* = 3). * *p* < 0.05 (one-way ANOVA, Tukey’s post hoc test).

The combination of NBs and US induces transient membrane permeabilization, significantly enhancing pDNA uptake and expression in cancer cells. We evaluated pDNA delivery in EBC2 tumor-bearing mice using luciferase-encoding pDNA delivered via NBs. After intratumoral injection, tumors were immediately exposed to US. Luciferase activity was measured 24 h post-treatment using an in vivo imaging system. Tumors were then harvested and analyzed via reporter assays. Figure 3 shows significantly elevated gene expression with combined NBs and pDNA administration plus US irradiation. NBs or US alone did not produce this effect, underscoring that NBs alone did not yield these results. This suggests the importance of NBs’ role in US-mediated gene delivery.

### 3.4. Evaluation of Target Gene Downregulation Using pCRISPRi Delivery via NBs and US

**Figure 4:**
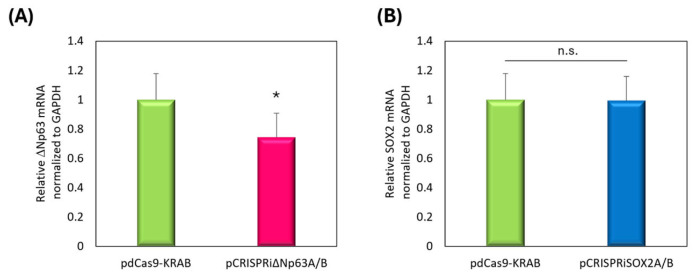
Real-time PCR analysis performed on day 6 following treatment with pCRISPRi, NBs, and US (frequency: 1 MHz; duty cycle: 50%; intensity: 2.0 W/cm^2^; duration: 2 min). Each sample (20 μg NBs + 10 μg pDNA) was intratumorally administered, followed by US on days 1, 3, and 5. (**A**) Relative mRNA expression levels of ΔNp63, normalized to GAPDH. (**B**) Relative mRNA expression levels of SOX2, normalized to GAPDH. Data: mean ± SD (*n* = 3). * *p* < 0.05 (Student’s *t*-test). n.s. indicates not statistically significant.

We previously reported that ΔNp63 or SOX2 may be effective in treating lung and esophageal SCC [11,35]. In CRISPRi-mediated suppression, dual gRNA expression systems targeting multiple promoter regions have shown improved transcriptional repression. Here, multiplex gene targeting was performed using dCas9-KRAB and tandem gRNA cassettes. As in Figure 3, we evaluated pCRISPRi delivery in EBC2 tumor-bearing mice via NBs and US. After intratumoral injection, tumors were immediately exposed to US. Tumors were then harvested 24 h post-treatment and analyzed mRNA level via real-time PCR. Figure 4A showed significant downregulation of ΔNp63 expression. Although SOX2 downregulation was not clearly observed in vivo, we detected upregulation of CDKN1A, a known downstream target of SOX2 (Supplementary Figure S1).

### 3.5. pCRISPRi-Induced Apoptosis

**Figure 5:**
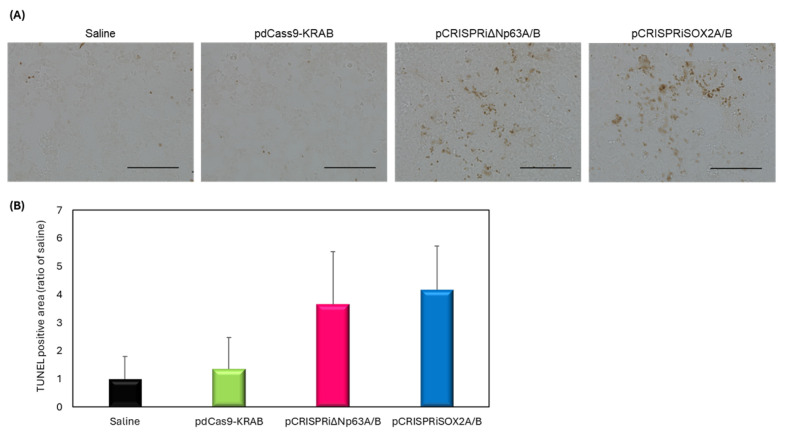
TUNEL staining post-treatment with pCRISPRi, NBs, and US (frequency: 1 MHz; duty cycle: 50%; intensity: 2.0 W/cm^2^; duration: 2 min). Each sample (20 μg NBs + 10 μg pDNA) was intratumorally administered, followed by US. (**A**) Representative images (scale bar = 100 μm). (**B**) TUNEL-positive area. Data: mean ± SD (*n* = 3).

Efficacy of pCRISPRi delivery post-tumor proliferation was assessed using TUNEL staining. Figure 5A displays representative TUNEL images. Figure 5B quantifies apoptotic cells. Figure 5B did not show statistically significant apoptosis induction. These results underscore the complexity of therapeutic timing and delivery efficiency, and suggest further investigation is needed.

### 3.6. Antitumor Effect of pCRISPRi via NBs and US Irradiation

**Figure 6:**
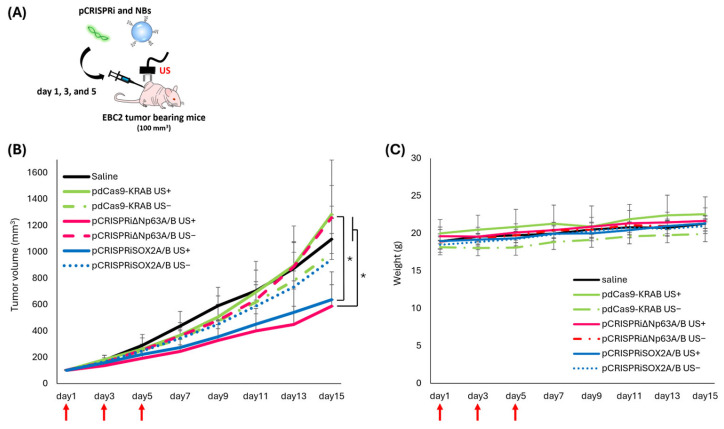
Local pCRISPRi delivery using NBs and US (frequency: 1 MHz; duty cycle: 50%; intensity: 2.0 W/cm^2^; duration: 2 min) thrice every other day. Each tumor received 20 μg NBs and 10 μg pDNA via intratumoral injection. Red arrows indicate the administration day. (**A**) Treatment schematic. (**B**) Tumor volume measured bi-daily until day 15. (**C**) Mouse body weight changes. Data: mean ± SD (*n* = 5). * *p* < 0.05 (one-way ANOVA, Tukey’s post hoc test).

We evaluated tumor growth suppression using CRISPRi targeting ΔNp63 or SOX2 in an EBC2 lung SCC xenograft model. Figure 6B shows significant tumor suppression in the US- and NB-treated group. No treatment-related side effects (e.g., weight loss) were observed (Figure 6C).

## 4. Discussion

Efficient transcriptional repression via CRISPRi typically depends on co-expression of two key elements: a catalytically inactive Cas9 fused to a repressor domain (e.g., KRAB) and a sequence-specific gRNA. To enable intracellular delivery, both components are frequently incorporated into a single plasmid vector. However, combining them increases plasmid size, which can negatively impact transfection efficiency, especially in non-viral delivery systems. Therefore, many studies turned to viral vectors for CRISPRi delivery, leveraging their high transduction efficiency. Nonetheless, viral approaches face limitations, including restricted packaging capacity, safety concerns, and potential host immune responses, which hinder clinical translation.

Non-viral vectors may overcome many of these limitations, particularly regarding safety, packaging capacity, repeated dosing, and in vivo applications. Our approach employs a simpler and more flexible delivery system—pCRISPRi—using cavitation with NBs and US. This strategy avoids the need for complex carrier design and permits the delivery of large genetic constructs without substantial size constraints.

In this study, we demonstrated proof-of-concept for cancer gene therapy using pCRISPRi with NBs and US. We utilized the EBC2 lung SCC xenograft model to assess whether pCRISPRi targeting ΔNp63 or SOX2 suppresses tumor growth in vivo. Prior work showed that transplantation of EBC2 cells treated with adenoviral vectors to suppress ΔNp63 and SOX2 markedly inhibited tumor growth compared to untreated cells [11,35]. Based on validation experiments on SOX2 and ΔNp63, therapy began only after tumors were established, emulating a clinically relevant scenario. Initiating treatment when tumors averaged approximately 100 mm^3^, pCRISPRi targeting ΔNp63 or SOX2 significantly slowed tumor progression and induced apoptosis (Figure 5 and Figure 6). Compared to both pdCas9-KRAB controls and non-US-irradiated groups, tumor growth was significantly suppressed, confirming target-specific effects. In the current treatment protocol, ΔNp63 showed significant downregulation in tumor tissue, while SOX2 showed no apparent inhibition (Figure 4). Regarding the functionality of the plasmid, our pCRISPRiΔNp63A/B has previously been shown to suppress the expression of the target gene, as reported in our earlier studies [11]. Furthermore, we demonstrated that pCRISPRiSOX2A/B effectively suppressed SOX2 expression in cultured cells (Supplementary Figure S2). These results indicate that our CRISPRi plasmid constructs are capable of effectively targeting the genes of interest. The discrepancy between in vitro and in vivo SOX2 mRNA repression might be explained by differences such as transcriptional kinetics and the tumor microenvironment. Although a detailed analysis of the inhibitory effect of SOX2 is needed, we were able to detect significant upregulation of CDKN1A (Supplementary Figure S1), a known downstream target of SOX2, suggesting that pCRISPRiSOX2 was functional upon our transfection. While the duration of inhibition and roles of each target factor also merit further evaluation, these results support that NBs and US deliver pCRISPRi to EBC2 tumors and successfully suppress SOX2 and ΔNp63, validating them as therapeutic targets in lung SCC.

CRISPRi is generally considered safer than RNAi or Cas9-based knockout approaches because it represses transcription through targeted binding of dCas9-KRAB to a specific region downstream of the transcription start site (TSS), and most off-target bindings outside this window are unlikely to elicit functional repression [40]. However, off-target gene repression remains a potential concern. To rigorously validate the specificity of our pCRISPRi system, further experiments such as ChIP sequencing, transcriptome-wide RNA sequencing, or qPCR analysis of predicted off-target genes should be performed in future studies. These analyses will help ensure that gene silencing is specifically directed to targeted genes and does not affect unintended genes.

Our NBs, characterized by uniform nanoscale dimensions via nanoparticle tracking analysis (Figure 1), show promise for spatially and temporally controlled gene delivery (Figure 3). Due to their nanoscale size, they can traverse intercellular gaps and penetrate deeply into tumor tissues, improving distribution for gene expression throughout the US-irradiated region (Figure 2).

Systemic administration also warrants consideration. Reports describe lung cancer diagnosis and treatment using systemically administered contrast agents [41,42]. Systemic delivery offers the advantage of reaching tumors that are difficult to directly assess and organs unsuitable for local delivery due to invasiveness. Compared to MBs, systemically administered NBs reportedly distribute widely through the body via microvessels when administered systemically [38]. Therefore, distribution via microvessels could expand gene delivery scope. Previously, we developed gene- or nucleic acid-loaded NBs for systemic delivery [37,43,44,45,46], demonstrating their utility. Future work aims to integrate NBs with gene- and nucleic acid-loading technologies, envisioning a theranostic platform that combines NBs and US for both therapeutic and diagnostic applications in cancer gene therapy compared to a combination of MBs and US.

US cannot penetrate healthy, air-filled lung tissues due to its scattering. Thus, applications of NBs and US in lung pathology are limited. However, ultrasonic waves can access fluid-filled regions in airway inflammation. Thoracic US is routinely used to evaluate pleural effusion [47]. Sugiyama et al. demonstrated that combining thoracic US and MBs selectively enhances drug delivery and cellular uptake in damaged lung tissue [48]. Although further validation using autologous transplantation is needed, US and NBs may allow for selective targeting of lung cancer with pleural effusion.

We previously reported that suppression of ΔNp63 and SOX2 is effective in inhibiting lung and esophageal SCC growth [11,35]. Esophageal SCC is also expected to be a promising target disease for this approach. When applying this method to esophageal SCC, it may be useful to irradiate near the diseased area using endoscopic US [49,50]. Moreover, the CRISPRi system can target multiple genes simply by changing the gRNA sequences. However, this study was limited to the EBC2 cell line, and further validation using other lung and esophageal SCC models is necessary to confirm the generalizability of these findings. In particular, evaluation in orthotopic transplantation models would help elucidate therapeutic effects under physiologically relevant microenvironments. It is anticipated that this research will contribute to the development of therapy not only for lung SCC but also for other challenging malignancies.

## 5. Conclusions

This study strongly supports ΔNp63 and SOX2 as therapeutic targets for lung SCC. Utilizing a non-viral, NB- and US-mediated delivery platform, we achieved targeted delivery of pCRISPRi, resulting in significant tumor growth inhibition and apoptosis. Compared to traditional viral systems, our approach offers improved safety, flexibility, and the capacity for large genetic constructs. The NBs-US combination provides spatiotemporally controlled gene delivery and is potentially adaptable to other cancers via simple gRNA modification. This result suggests that the NBs/US-mediated CRISPRi platform may be useful as an effective gene-targeted cancer therapy.

## Figures and Tables

**Table 1:** Characterization of NBs.

Mean Size (nm)	Span (nm)	Concentration (×1011 Particles/mL)	Zeta Potential (mV)
198.0 ± 17.9	1.47 ± 0.09	1.65 ± 0.38	−20.0 ± 0.41

Each value represents the mean ± standard deviation of three independently prepared samples (*n* = 3).

## Data Availability

The original contributions presented in this study are included in the article/Supplementary Materials. Further inquiries can be directed to the corresponding authors.

## Supplementary Materials

The following supporting information can be downloaded at: https://www.mdpi.com/article/10.3390/pharmaceutics17081053/s1, Video S1: Multi-laser nanoparticle tracking analysis visualization of NBs under simultaneous multi-laser illumination; Supplementary Figure S1: Effects on the expression levels of CDKN1A by the transfection of pCRISPRiSOX2A/B in vivo; Supplementary Figure S2: Downregulation of SOX2 by the transfection of pCRISPRiSOX2A/B in vitro.

## Abbreviations

The following abbreviations are used in this manuscript:

SCC	squamous cell carcinoma
CRISPRi	CRISPR interference
NBs	nanobubbles
US	ultrasound
