# Supplementary material for: Therapeutic pCRISPRi Delivery to Lung Squamous Cell Carcinoma by Combining Nanobubbles and Ultrasound
Source: Pharmaceutics. 2025 Aug 13;17(8):1053. doi: 10.3390/pharmaceutics17081053 (PMC12388912; doi:10.3390/pharmaceutics17081053)
Supplement: Supplementary file 1 [file pharmaceutics-17-01053-s001.zip › Supplementary Figure Legends.pdf]

Article

Therapeutic pCRISPRi Delivery to Lung Squamous Cell Carcinoma by Combining Nanobubbles and Ultrasound

Taiki Yamaguchi [1], Yoko Endo-Takahashi [1*], Takumi Amano [1], Arina Ihara [1], Tetsushi Sakuma [2], Takashi Yamamoto [3], Takuya Fukazawa [4], Yoichi Negishi [1*]

Video S1

Multi-laser nanoparticle tracking analysis visualization of NBs under simultaneous multi-laser illumination

Supplementary Figure S1

Effects on the expression levels of CDKN1A by the transfection of pCRISPRiSOX2A/B in vivo.

Real time PCR analysis performed on day 6 following treatment with pCRISPRiSOX2A/B, NBs, and US (frequency: 1 MHz, duty cycle: 50%; intensity: 2.0 W/cm2, duration: 2 min). Each sample (20 μg NBs + 10 μg pDNA) was intratumorally administered, followed by US on days 1, 3, and 5. Relative mRNA expression levels of CDKN1A, normalized to GAPDH. Data: mean ± SD (n = 3). * $p < 0.05$ (Student's t-test).

Supplementary Figure S2

Downregulation of SOX2 by the transfection of pCRISPRiSOX2A/B in vitro.

One day before transfection, the EBC2 cells were seeded into 24-well plates to a density of 5×10^4 per well. Transfections were carried out using Lipofectamine 3000 (Thermo Fisher Scientific) according to the manufacturer's protocol. Transfected cells were harvested after an additional 48 h. Relative mRNA expression levels of SOX2, normalized to GAPDH, were measured by real time PCR. Data: mean ± SD (n = 5). * $p < 0.05$ (Student's t-test).
