# Supplementary figures and images for: Therapeutic pCRISPRi Delivery to Lung Squamous Cell Carcinoma by Combining Nanobubbles and Ultrasound
Source: Pharmaceutics. 2025 Aug 13;17(8):1053. doi: 10.3390/pharmaceutics17081053 (PMC12388912; doi:10.3390/pharmaceutics17081053)

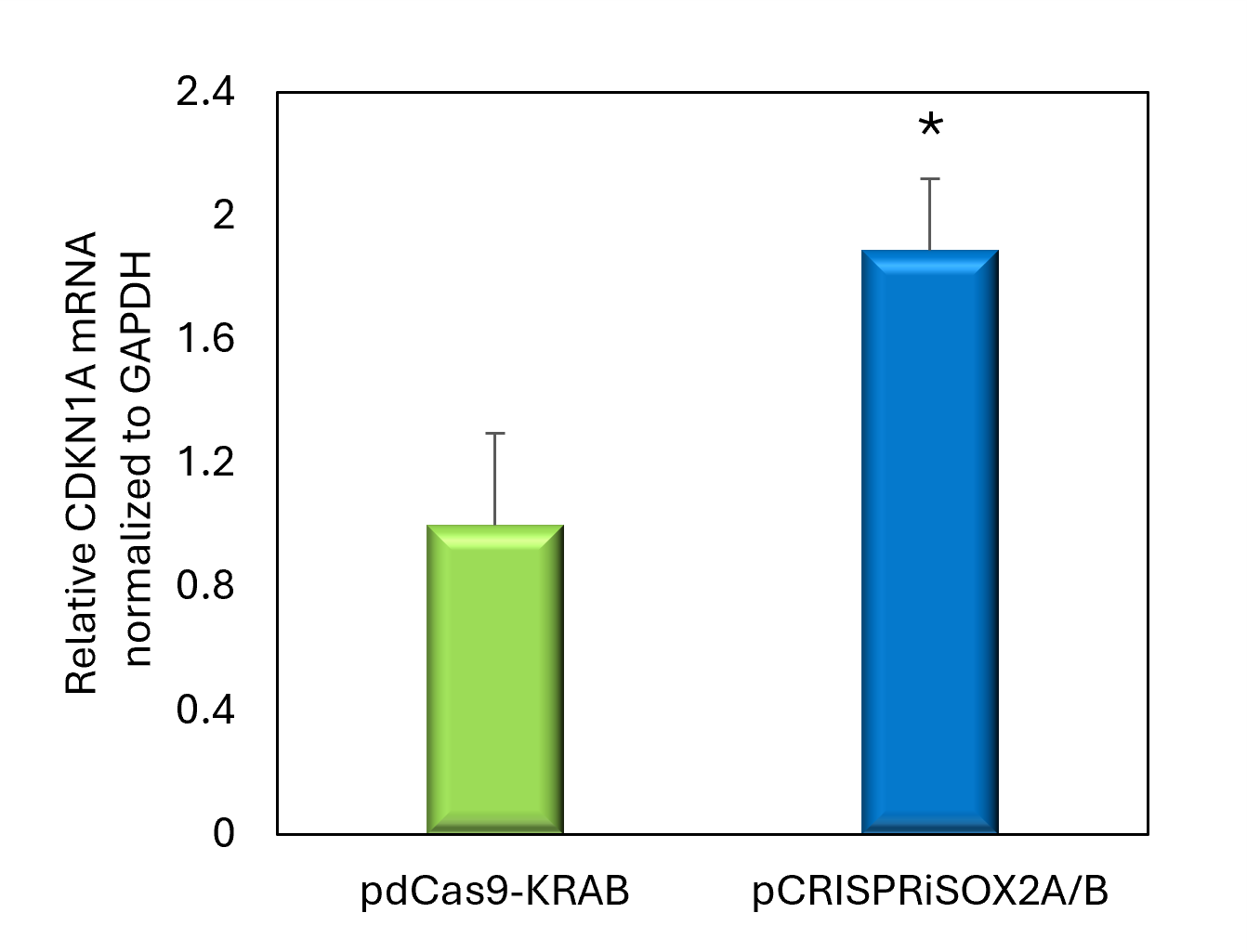

Supplement: Supplementary file 1 [file pharmaceutics-17-01053-s001.zip › Figure S1.tif]

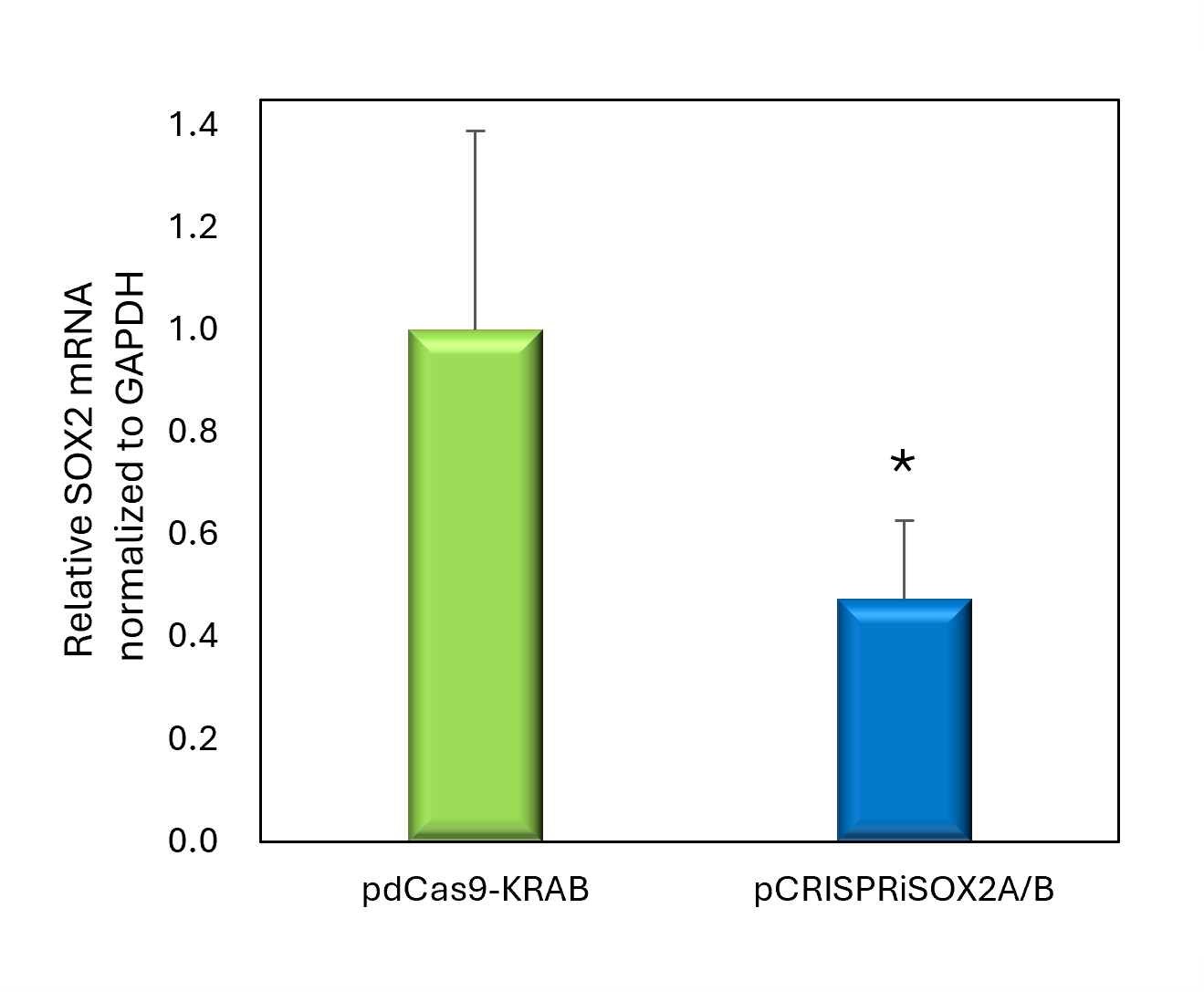

Supplement: Supplementary file 1 [file pharmaceutics-17-01053-s001.zip › Figure S2.tif]
